# Investigation of Piezoelectric Properties in Ca-Doped PbBa(Zr,Ti)O3 (PBZT) Ceramics

**DOI:** 10.3390/mi15081018

**Published:** 2024-08-09

**Authors:** Jolanta Makowska, Marian Pawełczyk, Andrzej Soszyński, Tomasz Pikula, Małgorzata Adamczyk-Habrajska

**Affiliations:** 1 Institute of Materials Engineering, Faculty of Science and Technology, University of Silesia, 75 Pułku Piechoty 1A, 41-500 Chorzow, Poland; jolanta.makowska@us.edu.pl; 2 Institute of Information Technologies, Mickiewicza 29, 40-085 Katowice, Poland; rektor@wsti.pl; 3 Institute of Physics, University of Silesia, ul. 75 Pułku Piechoty 1, 41-500 Chorzow, Poland; andrzej.soszynski@us.edu.pl; 4 Institute of Electronics and Information Technology, University of Technology, 38A Nadbystrzycka Str., 20-618 Lublin, Poland; t.pikula@pollub.pl

**Keywords:** PBZT, piezoelectric properties, calcium dopant

## Abstract

The perovskite-structured materials ${\left({Pb}_{0.75}{Ba}_{0.25}\right)}_{1-x}{Ca}_{x}({Zr}_{0.7}{Ti}_{0.3})O_{3}$ for *x* = 1 and 2 at.% were synthesized using the conventional mixed-oxide method and carbonates. Microstructural analysis, performed using a scanning electron microscope, revealed rounded grains with relatively inhomogeneous sizes and distinct grain boundaries. X-ray diffraction confirmed that the materials exhibit a rhombohedral structure with an *R*3*c* space group at room temperature. Piezoelectric resonance measurements were conducted to determine the piezoelectric and elastic properties of the samples. The results indicated that a small amount of calcium doping significantly enhanced the piezoelectric coefficient d_31_. The calcium-doped ceramics exhibited higher electrical permittivity across the entire temperature range compared to the pure material, as well as a significant value of remanent polarization. These findings indicate that the performance parameters of the base material have been significantly improved, making these ceramics promising candidates for various applications.

## 1. Introduction

Materials based on lead zirconium titanate (PZT) are the main building blocks of electro-electronic devices such as high-energy capacitors, non-volatile memories (FRAM), ultrasonic sensors, infrared detectors, etc. [1,2,3,4,5]. Ceramics exhibiting the characteristics of ferroelectric relaxors occupy a particularly important place in this group of materials [6,7,8,9,10]. These materials, known for their disordered structures and unique properties, transition from a non-polar paraelectric phase at high temperatures to an ergodic relaxor state characterized by nanometer-scale polar regions with randomly oriented dipole moments as they cool to the Burns temperature (T_B_) [11]. These unique properties of ferroelectric relaxors are exploited, among others, in electro-optic devices [12]. Their ability to change refractive indices in response to an electric field makes them useful in modulators, switches, and other components in optical communication systems. Due to their strong piezoelectric properties, ferroelectric relaxors are also suitable for piezoelectric devices, including ultrasonic transducers, medical imaging equipment, and acoustic sensors [13,14,15,16]. Their high sensitivity and broad operational temperature range provide advantages over traditional piezoelectric materials.

Relaxor behavior was initially observed in perovskites with disordered non-isovalent ions, such as stoichiometric complex perovskite compounds and nonstoichiometric solid solutions derived from the PZT system [17]. It is well-established that the properties of PZT can be significantly improved by introducing various dopants into the “A” or “B” sites [17]. Such additives induce relaxor behavior [18,19], with barium ions being a prime example. The Ba-modified Pb(Zr_1−x_Ti_x_)O_3_ (PBZT) ceramic composition has long been an interesting ferroelectric material due to its remarkable physical properties, including a very high electrical permittivity that exhibits weak temperature dependence [20,21,22]. Research on the PBZT ceramic composition began with Smolenskii et al. [23], while structural analyses were conducted by Ikeda [24], who developed the phase diagram accordingly. Subsequent researchers discovered [25] that ceramics within the compositional boundaries of the ferroelectric (FE) rhombohedral, tetragonal, and paraelectric (PE) cubic phases exhibit characteristics typical of a ferroelectric relaxor.

One of the representatives of this type of material is Pb_0.75_Ba_0.25_Zr_0.70_Ti_0.30_ ceramic. Its properties, including, in particular, the characteristics typical of ferroelectric relaxors, as well as its piezoelectric properties, have been extensively discussed in our previous publications [26,27,28,29].

However, work is still ongoing to improve the application properties of this material, including enhancing its piezoelectric parameters. It is widely known that materials with better density and fewer pores exhibit better piezoelectric properties. This effect can be achieved, among other methods, through appropriate doping. It is well known that calcium ions introduced into the structure of classical perovskite, such as barium titanate, improve its sinterability and, consequently, positively affect its application parameters, including its piezoelectric coefficients [30,31]. Based on this information, we decided to use calcium ions as modifiers of PBZT 25/70/30 ceramics. The present paper describes the influence of calcium dopants on the microstructure and piezoelectric properties of the discussed materials, focusing on potential improvements in their performance for practical applications. This is a novel aspect of the present work, as the influence of calcium ions on the piezoelectric properties of PBZT materials with compositions near the morphotropic phase boundary has not been previously studied. There are also no reports on their impact on ferroelectric properties, including the value of the remanent polarization.

## 2. Materials and Methods

Powders with specific compositions were prepared using stoichiometric mixtures of the primary oxides or carbonates: PbO, BaCO_3_, ZrO_2_, TiO_2_, and CaCO_3_. These ingredients were mixed for t = 12 h, then compressed into cylindrical pellets and subjected to a thermal synthesis process at a temperature of T = 925 °C for a duration of t = 2 h. The appropriate quantities of reagents were weighed according to the following Formula (1):

(1) $$0.75\left(1-x\right)PbO+0.25\left(1-x\right){BaCO}_{3}+x{CaCO}_{3}+0.7{ZrO}_{2}+0.3{TiO}_{2}{\left({Pb}_{0.75}{Ba}_{0.25}\right)}_{1-x}{Ca}_{x}({Zr}_{0.7}{Ti}_{0.3})O_{3}$$

Next, the powders were crushed, ground and sieved, compressed into cylindrical pellets, and sintered at T = 1250 °C for t = 4 h. This step was repeated before the final sintering, conducted at T = 1300 °C for t = 12 h. To maintain the established composition and, in particular, to avoid loss of PbO due to sublimation, the sintering processes were carried out in a crucible with the addition of a certain amount of PbO and ZrO_2_.

The microstructure was analyzed using a scanning electron microscope (JEOL JSM-7100F TTL LV, Akishima, Japan) with an energy-dispersive spectrometer (EDS). Qualitative and quantitative evaluations of the chemical composition were conducted using the X-ray microanalysis technique.

The bulk density of the discussed materials was measured using Archimedes’ method with water as the immersing medium.

X-ray diffraction (XRD) measurements were carried out using a Huber diffractometer with θ–2θ geometry. Each pattern was measured from 19° to 100° in 2θ with a step size of 0.02° and analyzed using a set of programs, i.e., the DHN powder diffraction system ver. 2.3.

The investigations of the hysteresis loops were carried out using the computerized automatic modified Diamant, Drenck, and Pepinsky measuring system. The measuring system made it possible to determine the values of the remanent spontaneous polarization (Pr) on the basis of the observed saturated hysteresis loops.

Dielectric measurements were performed using an LCR meter Agilent E4980A (Santa Clara, CA, USA) (at frequencies of the measurement field from 0.1 kHz to 1.0 MHz at the heating cycle).

For measurements of temperature changes in electrical permittivity and the tangent of the loss angle, as well as for measurements of the electrical hysteresis loop, disk-shaped samples with a surface area of 1 cm^2^ and thicknesses of 0.6 mm and 0.3 mm, respectively, were used. The cut and polished samples were coated with silver electrodes, using a silver paste without thermal treatment.

Piezoelectric properties were determined based on measurements of piezoelectric resonance [32,33,34]. The measurements were undertaken using a Hewlett-Packard 4192A (Palo Alto, CA, USA) impedance analyzer on samples prepared in a bar shape with dimensions of about 4 × 1 × 0.5 mm^3^ [29]. The dimensions of the used samples were in accordance with the geometric conditions imposed by the authors of this paper [34]. According to these authors, the geometric conditions for samples intended for determining the piezoelectric coefficient d_31_ are as follows:

$${\left(\frac{l}{w}\right)}^{2}\geq 10\;\mathrm{and}\;{\left(\frac{l}{a}\right)}^{2}\geq 10$$

where *l*, *w*, and *a* are the length, width, and height of the sample.

The respective surfaces were coated with gold electrodes. Electrical contact with the measuring system was made by means of thin gold wires glued to the centers of the electrodes. The samples were poled using an electrical field of 6 kV/cm applied at 473 K for 20 min and then slowly cooled to room temperature.

## 3. Results

### 3.1. Scanning Electron Microscopy and X-ray Microanalysis (EDS) 

**Figure 1:**
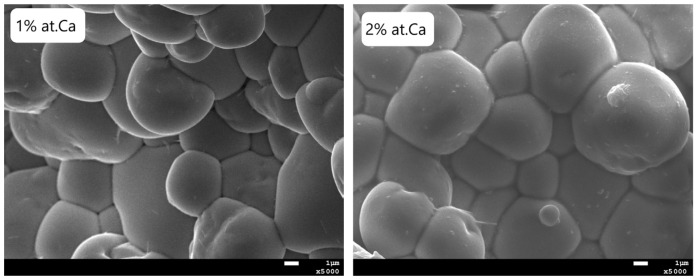
Microstructure of PBZT ceramics modified by 1 and 2 at.% of Ca.

Figure 1 shows the microstructure of PBZT ceramics modified by 1 and 2 at.% of Ca. Analyzing the presented images, it can be clearly stated that the microstructure is characterized by rounded grains that are relatively heterogeneous in size, densely packed, and without visible pores between them. For all images discussed, the boundaries between grains are clearly marked. This indicates that adding calcium may help control grain growth during sintering. The average size of grains is equal 13.5 μm and 11 μm for 1 at.% and 2 at.% of calcium dopant.

**Figure 2:**
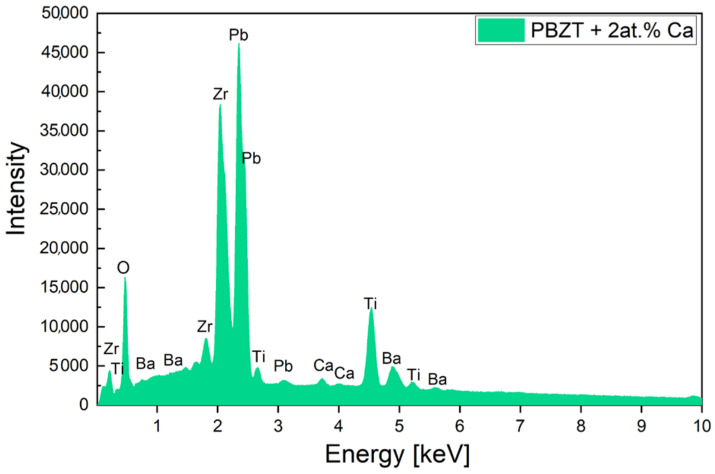
Exemplary EDS spectrum on the PBZT ceramics modified by 2 at.% of Ca.

The ceramic materials were subjected to energy-dispersive X-ray (EDS) analysis. Figure 2 displays an exemplary EDS spectrum for the PBZT ceramics modified by 2 at.% of Ca. The results confirmed the absence of any extraneous elements or impurities.

Quantitative analysis of the chemical composition was carried out in 50 randomly selected micro-areas of each of the considered ceramic materials to determine the degree of deviation of the actual elemental content from the theoretical stoichiometry (Table 1). Then, the average values were calculated. The variation between the average percentage contents of the individual components of the discussed compounds and the theoretical stoichiometry is small and falls within the error margin of the applied method.

The bulk density determined by Archimedes’ method is equal to 6925 and 6954 [kg/m^3^], respectively, for 1 at.% and 2 at.% of calcium dopant. These values are higher compared to the density of undoped PBZT 25/70/30 ceramics, which is equal to 6800 [kg/m^3^] [35].

**Figure 3:**
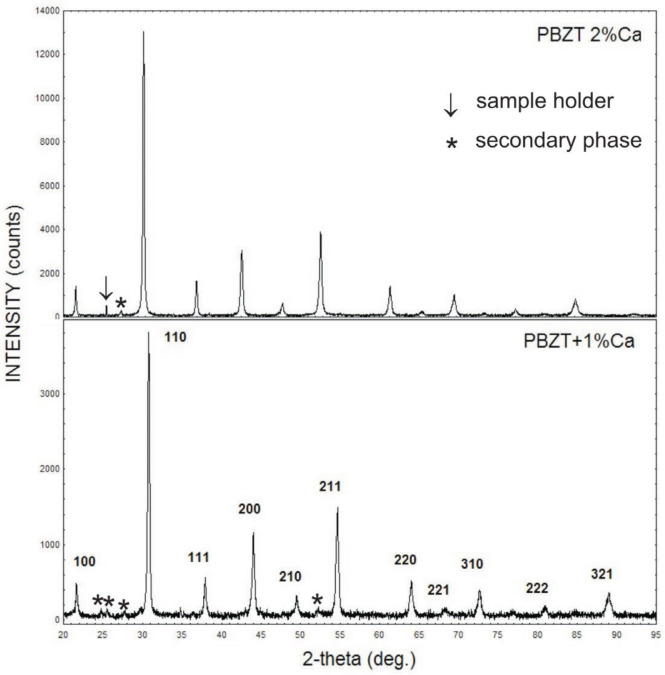
X-ray diffraction pattern (XRD) of calcium-doped PBZT ceramics.

As mentioned above, XRD examined the crystallographic structure of the ceramics. The X-ray diffraction patterns (XRD) of calcium-doped PBZT ceramics obtained at room temperature are shown in Figure 3.

The main peaks of diffractograms were indexed according to a rhombohedral, perovskite-like ABO_3_ structure of space group R3c. This agrees well with the data reported earlier by Mir et al. [36]. However, the departure of the lattice from cubic symmetry is very little. The parameters of the unit cell are equal a = 4.1140 (4) (Å), = 89.969 [deg] for ceramics with an admixture of 1 at.% of calcium and a = 4.1115 (1) (Å), α = 89.968 [deg] for ceramics with 2 at.% of Ca, respectively. The lattice parameters are slightly smaller compared to those for a pure PBZT 25/70/30 material, which is a result of differences in the size of the ionic radius of lead, barium, and calcium. Moreover, the small admixture of Ca significantly enhanced the piezoelectric d_31_ coefficient, as is shown in Section 3.4. Thus, it can be claimed that Ca ions were incorporated into the rhombohedral lattice of PBZT. A tiny amount of secondary phase/phases was detected (peaks marked by a star in Figure 3). In fact, it is impossible to identify the phase the peaks come from, as their signal is close to the XRD detection limit. In the case of the sample with an admixture of 2 at.% of Ca, a weak signal from the sample holder can also be seen. This is evidenced by the narrow peak close to 25.5 [deg].

### 3.2. Dielectric Properties

**Figure 4:**
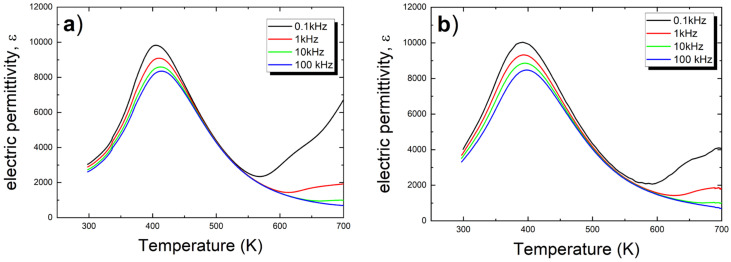
Temperature dependencies of the dielectric constant measured on heating at various frequencies of measuring field for calcium-modified ceramics (**a**) 1 at.% (**b**) 2 at.%.

The measurement of the temperature dependence of electrical permittivity and the tangent of the loss angle was conducted during the heating process in the temperature range from room temperature to 700 K. The characteristics obtained at four different measurement field frequencies are presented in Figure 4 for both discussed materials.

Note that for both discussed ceramic materials, the value of permittivity is significantly higher compared to the undoped material over the entire considered temperature range—Table 2. Furthermore, the concentration of calcium ions also affects the value of electrical permittivity. It is also worth mentioning that the addition of calcium lowers the temperature at which the maximum permittivity T_m_ occurs. The temperature characteristics ε(T) exhibit frequency dispersion (see Figure 4 and Table 2), which is a characteristic feature of ferroelectric relaxors, and was described in more detail in our previous publication. This dispersion gradually decreases with the increase in the amount of calcium ions, as evidenced by the reduction in the values of the dispersion coefficients for both the temperature T_m_ (ΔT_m_) and the maximum value of electrical permittivity ε_max_ (Δε_max_). The coefficient ΔT_m_ is defined as the difference between the T_m_ measured at 0.1 kHz and that measured at 100 kHz. The coefficient Δε_max_ is defined in a similar manner.

**Figure 5:**
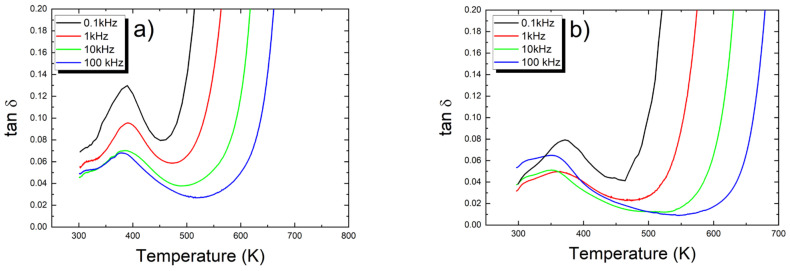
Temperature dependencies of the loss factor measured on heating at various frequencies of measuring field for calcium-modified ceramics (**a**) 1 at.% (**b**) 2 at.%.

The temperature dependencies of the loss factor (tanδ) of both discussed ceramics are presented in Figure 5.

The loss factor also strongly depends on the frequency of the measuring electric field. The temperatures corresponding to ε_max_ and tanδ_max_ are fairly consistent, whereas the local minima in the tanδ (T) curves occur at temperatures higher than T_m_, which contrasts with classical ferroelectrics. The value of the loss factor, both at room temperature and at its maximum, does not change significantly compared to the undoped ceramics (see Table 2).

### 3.3. Hysteresis Loop Measurements

**Figure 6:**
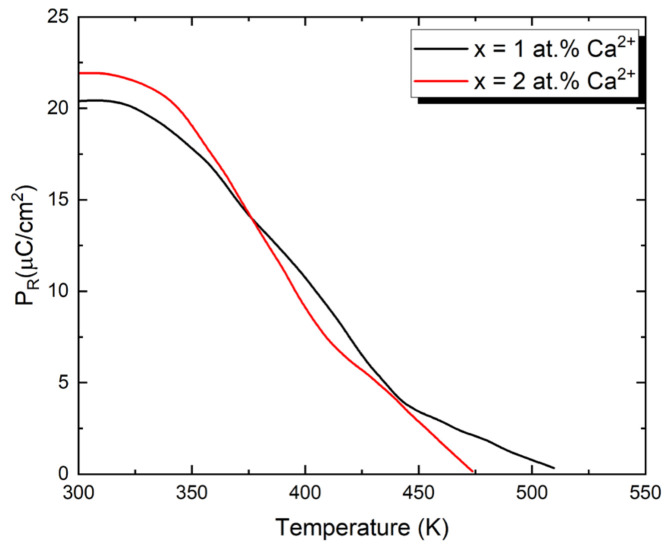
The remanent polarization of calcium ions modified PBZT 25/70/30 ceramics as a function of temperature, obtained from hysteresis loop measurements.

The temperature dependence of remanent polarization (P_R_) determined on heating from the hysteresis loop measurements is shown in Figure 6. These measurements were carried out in a field of frequency 50 Hz and strength 10 kV/cm. The shape of the presented dependencies P_R_(T) is typical for ferroelectric relaxors. Namely, the maximum of polarization appears at temperatures far below T_m._ The maximum value of P_R_ slightly depends on the dopant ion content, increasing as their concentration rises. It is also worth noting that the maximum remanent polarization (P_Rmax_) for both considered ceramic materials significantly exceeds the P_Rmax_ value of the undoped ceramics, which is equal to 6.5 μC/cm^2^ [27].

### 3.4. Piezoelectric Properties

**Figure 7:**
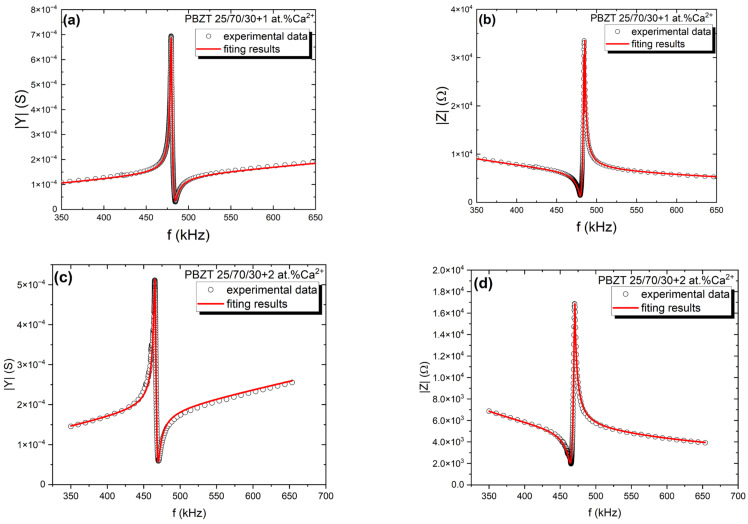
Frequency dependencies of (**a**,**c**) the modulus |Y | of the complex admittance *Y = G + iB* and (**b**,**d**) the modulus |Z| of the complex impedance *Z = R + iX*.

In aiming to obtain the d_31_ piezoelectric coefficient, the modulus admittance |Y| and phase angle θ were measured in the function of frequency. The experimental results allowed us to determine the frequency dependencies of the impedance modulus |Z|. The frequency’s dependencies of both moduli for all modified ceramics are shown in Figure 7. The maximum and minimum of |Y(f)|correspond to the resonance f_r_ and antiresonance f_a_ frequency, respectively.

On the other hand, following the method widely described by the authors of [32], the complex admittance is written as Equation (2):

(2) Y=G+iB

where *G* and *B* are, respectively, the real and imaginary parts of *Y*. The real part of admittance in turn is given by theoretical Equation (3):

(3) $$G\left(\omega \right)=\beta \frac{\omega^{2}\Gamma }{{\left(\omega_{r}^{2}-\omega^{2}\right)}^{2}+\omega^{2}\Gamma^{2}}+C_{0}^{\prime \prime }\omega$$

whereas the equation that describes the imaginary part of admittance has the following form (4):

(4) $$B\left(\omega \right)=\beta \frac{{\left(\omega_{r}^{2}-\omega^{2}\right)}^{2}\omega }{{\left(\omega_{r}^{2}-\omega^{2}\right)}^{2}+\omega^{2}\Gamma^{2}}+C_{0}^{\prime }\omega$$

So, the modulus of admittance |Y| is described by Equation (5):

(5) $$\left|Y(\omega )\right|=\sqrt{G^{2}\left(\omega \right)+B^{2}\left(\omega \right)}$$

(3), (4) →(5)

(6) $$\left|Y(\omega )\right|=\sqrt{{\left(\beta \frac{\omega^{2}\Gamma }{{\left(\omega_{r}^{2}-\omega^{2}\right)}^{2}+\omega^{2}\Gamma^{2}}+C_{0}^{\prime \prime }\omega \right)}^{2}+{\left(\beta \frac{{\left(\omega_{r}^{2}-\omega^{2}\right)}^{2}\omega }{{\left(\omega_{r}^{2}-\omega^{2}\right)}^{2}+\omega^{2}\Gamma^{2}}+C_{0}^{\prime }\omega \right)}^{2}}$$

where ω_r_ = 2πf_r_ and ω_a_ = 2πf_a_.

In a similar manner, the complex of impedance is described as follows (7):

(7) Z=R+iX

where *R* and *X* are real and imaginary parts of the impedance, respectively. Their frequency dependencies are given by the following, Equations (8)–(10):

(8) $$R\left(\omega \right)=\alpha \frac{\Gamma }{{\left(\omega_{a}^{2}-\omega^{2}\right)}^{2}+\omega^{2}\Gamma^{2}}+R_{0}$$

(9) $$X\left(\omega \right)=\alpha \frac{\left({\omega^{2}-\omega }_{a}^{2}\right)\left(\omega -\frac{\omega_{r}^{2}}{\omega }\right)+\Gamma^{2}}{{\left(\omega_{a}^{2}-\omega^{2}\right)}^{2}+\omega^{2}\Gamma^{2}}$$

(10) $$\left|Z(\omega )\right|=\sqrt{R^{2}\left(\omega \right)+X^{2}\left(\omega \right)}$$

(11) $$\left|Z(\omega )\right|=\sqrt{{\left(\alpha \frac{\Gamma }{{\left(\omega_{a}^{2}-\omega^{2}\right)}^{2}+\omega^{2}\Gamma^{2}}+R_{0}\right)}^{2}+{\left(\alpha \frac{\left({\omega^{2}-\omega }_{a}^{2}\right)\left(\omega -\frac{\omega_{r}^{2}}{\omega }\right)+\Gamma^{2}}{{\left(\omega_{a}^{2}-\omega^{2}\right)}^{2}+\omega^{2}\Gamma^{2}}\right)}^{2}}$$

In the next step, the experimentally measured *|Y|(f)* and *|Z|(f)* frequency dependencies were fitted to the theory given by the sets of Equations (6) and (10). The red line on the dependencies in Figure 7 is a fit for the experimental data (black points). The fitting procedure of the *|Z|(f)* dependence allows us to determine the resonance and antiresonance frequencies, whereas the fitting procedure of *|Y|(f)* gives as the permittivity *ε_33_* along the *z* direction [32]. This is based on the parameters mentioned above and used the following, Equations (12)–(15):

(12) $$s_{11}^{\prime }=\frac{1}{4\rho l^{1}f_{r}^{2}}$$

(13) $$s_{11}^{\prime \prime }=\frac{\gamma }{f_{r}}s_{11}^{\prime }$$

(14) $$\frac{tan\left(\frac{\pi f_{a}}{2f_{r}}\right)}{\frac{\pi f_{a}}{2f_{r}}}=\frac{k_{31}^{2}-1}{k_{31}^{2}}$$

(15) $$d_{31}^{2}=\varepsilon_{0}\varepsilon_{33}s_{11}^{\prime }k_{31}^{2}$$

we calculated the piezoelectric coefficient d_31_, the complex elastic compliance (s′_11_ and s”_11_), and the coefficient of electromechanical coupling k_31_, measuring the ceramics density. The estimated parameters are collected in Table 3.

Calcium ions introduced into the crystal lattice of PBZT 25/70/30 ceramics cause a significant increase in the d_31_ coefficient as well as in the electromechanical coupling coefficient, making the discussed materials more attractive for electronic applications.

## 4. Conclusions

${\left({Pb}_{0.75}{Ba}_{0.25}\right)}_{1-x}{Ca}_{x}({Zr}_{0.7}{Ti}_{0.3})O_{3}$ for x = 1 and 2 at.% materials were prepared by a conventional mixed-oxide method. The X-ray diffraction studies proved the formation of rhombohedrally distorted perovskite-like lattices characterized by an *R*3*c* space group and the incorporation of Ca ions into the structure of PBZT. The test results described above clearly indicate the positive impact of calcium ions on the functional properties of PBZT 25/70/30 ceramics. The addition of a small amount (up to 2% at.) of this modifier increased the density of the tested ceramics, which, among other things, increases the hardness of the grains. Breakthroughs occur through grain boundaries and not inside the grains. The improvement of mechanical properties also translates into an increase in piezoelectric parameters, including the d31 coefficient. Moreover, ceramics doped with calcium ions show higher electrical permittivity in the entire temperature range compared to the pure material. It is worth emphasizing that a fivefold increase in permittivity at room temperature does not significantly change the value of the loss angle tangent. It remains small up to high temperatures (for a frequency of 0.1 kHz, the temperature is about 450 K), then its value begins to increase rapidly. However, this is beyond the scope of use of standard electronic devices. Therefore, as expected, the performance parameters of the base material have been significantly improved.

## Figures and Tables

**Table 1:** Theoretical and experimental comparison of the content of substrates forming the ${\left({Pb}_{0.75}{Ba}_{0.25}\right)}_{1-x}{Ca}_{x}({Zr}_{0.7}{Ti}_{0.3})O_{3}$ ceramics, converted to oxides.

Formula	Oxide Content by EDS Measurement [%]					Theoretical Content of Oxides [%]				
	PbO	BaO	CaO	ZrO_2_	TiO_2_	PbO	BaO	CaO	ZrO_2_	TiO_2_
PBZT + 1 at.%Ca	52.7	12.07	0.18	27.43	7.62	53.02	11.95	0.16	27.23	7.64
PBZT + 2 at.%Ca	52.42	12.00	0.36	27.56	7.66	51.95	12.9	0.35	28.25	6.55

**Table 2:** The dielectric parameters important for applications: the electric permittivity at room temperature (ε_RT_), the maximum value of electric permittivity (ε_max_), the temperature of electric permittivity maximum (T_m_), the loss factor at room temperature (tg_RT_), the maximum value of the loss factor (tgδ_max_), the dispersion coefficient of electric permittivity (Δε_max_), and the dispersion coefficient of T_m_ temperature (ΔT_m_).

	ε_RT_	ε_max_	T_m_	tgδ_RT_	tgδ_max_	Δε_max_	ΔT_m_
PBZT + 0 at.%Ca [c]	430	5390	464	0.02	0.06	1420	14.1
PBZT + 1 at.%Ca	2897	9089	410	0.05	0.09	1111	12.68
PBZT + 2 at.%Ca	3688	9323	393	0.04	0.05	1033	9.46

**Table 3:** Piezoelectric, dielectric, and elastic coefficients of PBZT and calcium-doped PBZT ceramics.

Sample	ε_33_	s′_11_[m^2^/N]	s″_11_[m^2^/N]	k_31_	d_31_[C/N]
PBZT 25/70/30 [29]	433.4	1.32 × 10^−11^	3.79 × 10^−14^	0.08	1.80 × 10^−11^
PBZT 25/70/30 + 1 at.% Ca^2+^	1042.7	1.09 × 10^−11^	2.19 × 10^−14^	0.16	4.92 × 10^−11^
PBZT 25/70/30 + 2 at.% Ca^2+^	1443.6	1.15 × 10^−11^	7.86 × 10^−14^	0.16	6.09 × 10^−11^

## Data Availability

The data will be made available on request.
